# Editorial: New-generation vaccines and novel vaccinal strategies against infectious diseases of livestock, wild and companion animals

**DOI:** 10.3389/fimmu.2023.1256363

**Published:** 2023-08-02

**Authors:** Brad Pickering, Raúl Manzano-Román, Suresh Kumar Tikoo, Christophe Chevalier, Denis Archambault

**Affiliations:** ^1^ National Centre for Foreign Animal Disease, Canadian Food Inspection Agency, Winnipeg, MB, Canada; ^2^ Department of Medical Microbiology and Infectious Diseases, College of Medicine, Faculty of Health Sciences, University of Manitoba, Winnipeg, MB, Canada; ^3^ Infectious and Tropical Diseases Group (e-INTRO), Institute of Biomedical Research of Salamanca-Research Center for Tropical Diseases at the University of Salamanca (IBSAL-CIETUS), Faculty of Pharmacy, University of Salamanca, Salamanca, Spain; ^4^ Vaccinology & Immunotherapeutics Program School of Public Health, and VIDO, University of Saskatchewan, Saskatoon SK, Canada; ^5^ INRAE, UVSQ, UMR892 VIM, Equipe Influenza Virus, Université Paris‐Saclay, Jouy‐en‐Josas, France; ^6^ Department of Biological Sciences, University of Quebec at Montreal, Montreal, QC, Canada

**Keywords:** infectious disease, fish, animals, vaccines, VLP/RNA/DNA-based vaccines, nanovaccine, immunogens/antigens, adjuvant

Vaccination against infectious disease is an invaluable tool to protect humans against severe morbidity and mortality. For this reason, significant advances in human vaccines have propelled the field of vaccinology forward. Emerging and neglected diseases still pose an important challenge (1); fortunately, the evolution of technology in the vaccinology field is providing modern options to successfully prevent viral and non-viral human infections (2, 3). In contrast, the development of animal vaccines has lagged, although their importance is just as critical to the health and welfare of wild, domestic, and companion animals. In addition to the zoonotic risk it poses to public health, infectious animal diseases have accounted for more than 20 billion euros in direct losses over the last decade, and more than ten times that amount in indirect costs (4). This Research Topic “*New-generation vaccines and novel vaccinal strategies against infectious diseases of livestock, wild and companion animals*” highlights advances and innovations in animal vaccines.

Within this Research Topic, both original research and review articles are presented. The original article “Targeted delivery of oral vaccine antigens to aminopeptidase N (APN) protects pigs against pathogenic *Escherichia coli* challenge infection” describes the complications of oral subunit vaccines and the significant hurdles in overcoming the barriers of the gastrointestinal tract, limiting their development and efficacy. However, by utilizing APN-specific antibody-antigen fusion constructs, researchers have demonstrated the induction of both mucosal and systemic immune responses in a piglet model of bacterial infection, providing a stepping stone toward the realization of an effective and protective oral subunit vaccine targeting APN. The manuscript by Souto et al. provides data to support the development of a bivalent vaccine candidate to protect fish from viral hemorrhagic septicemia (VHS) and viral encephalopathy and retinopathy (VER), major threats in aquaculture. By modifying the genome of viral hemorrhagic septicemia virus (VHSV) and introducing an expression cassette encoding the protective antigen domain of nervous necrosis virus (NNV) capsid protein, the authors successfully demonstrated the safety, immunogenicity, and protective efficacy of the recombinant VHSVs (rVHSV) in trout and sole. These findings hold promise for the development of a valuable bivalent live attenuated vaccine for commercially valuable fish species. Anotherstudy assessed the immune responses in calves to vaccines targeting *Mycobacterium avium* subspecies paratuberculosis (MAP), a cause of chronic enteritis in ruminants. Here, the authors analyzed the immune response induced by truncated MAP antigens as a fusion either on protein particles or as a soluble recombinant MAP (rMAP) fusion protein and compared this to a commercial vaccine. The rMAP fusion protein vaccine displayed the strongest immune response and showed promise in providing protective immunity against MAP infection while avoiding interference with bovine tuberculosis diagnostic tests. In another article, the authors describe a promising vaccination strategy for East Coast fever, a prevalent bovine disease in Africa caused by *Theileria parva*. In this study, using a recombinant lumpy skin disease virus (LSDV), the authors engineered virus-like particles (VLPs) containing a modified form of the *T. parva* p67 surface antigen and the bovine leukemia virus (BLV) gag gene. Studies in mice demonstrated the vaccine’s immunogenicity, showing higher antibody titers in the group vaccinated with the recombinant LSDV. This encouraging progress paves the way for further investigations and potential applications of this dual vaccine candidate in cattle. Using recombinant bovine herpesvirus (BHV)-4 expressing nonstructural protein 5 (NSP5) and M fusion protein of porcine reproductive and respiratory syndrome virus (PRRSV), a study suggested that a T cell response induced in recombinant viral vector primed pigs can help in reducing PRRSV-1–associated tissue damage without reducing the viral load. A separate study explored the potential of an adenoviral-vectored Epigraph vaccine as a promising alternative to current Swine Influenza A Virus (IAV-S) vaccines. Their findings demonstrated encouraging results, with the vaccine inducing robust and durable antibody responses in vaccinated pigs, as well as significant protection against viral challenge 6 months after initial vaccination.

To complement the original research outlined above, this Research Topic also delves into important questions regarding new vaccine strategies and considerations for future approaches in detailed reviews. “Recent advances in antigen targeting to antigen-presenting cells in veterinary medicine” outlines the dynamic field of veterinary medicine, where the quest for innovative strategies to combat challenging diseases has gained considerable momentum. Notably, groundbreaking advancements in antigen targeting, with a particular focus on antigen-presenting cells such as dendritic cells, through the use of DC peptides and MHC-II, have emerged as a beacon of hope. Moreover, another review describes the complications of vaccination in wildlife animals. Prion diseases, such as chronic wasting disease (CWD), pose significant challenges due to their unique biology and potential zoonotic risks. Current efforts to manage CWD have been largely ineffective, emphasizing the need for new tools such as vaccines. Despite the hurdles of overcoming immune tolerance and vaccinating wild animals, progress has been made in identifying safe antigens and effective strategies for formulation and delivery, including oral delivery to wild cervids.

The intricate immune system of the upper reproductive tract (URT) serves a remarkable purpose: shielding against sexually transmitted pathogens while simultaneously embracing immune tolerance toward sperm and the developing fetus. The review “Immune responses in the uterine mucosa: clues for vaccine development in pigs” explores the pursuit of effective strategies, with intrauterine immunization emerging as a promising approach, aiming to elicit localized or systemic immunity that safeguards against potential threats. Finally, Type I interferons (IFNs-α/β) are vital components of the innate immune response against viral infections. However, viruses have developed clever strategies to evade the antiviral effects of IFNs, compromising the efficiency of the immune system and vaccines. Understanding these evasion mechanisms can pave the way for the development of innovative vaccines that counteract viral IFN antagonism and induce robust immune responses for enhanced protection against a wide range of pathogens. The review article “Reprogramming viral immune evasion for a rational design of next-generation vaccines for RNA viruses” explores advances in developing IFN antagonism-deficient viruses, their immune evasion, and attenuated phenotypes in natural host animal species.

This Research Topic brings a diverse selection of topics outlining advances in the field of veterinary vaccinology. The importance of generating protective vaccines against disease in animals is critical to ensuring their health and wellness, thus favoring production systems and reducing zoonotic disease risk (5).

## Author contributions

BP: Writing – original draft, Writing – review & editing. RM-R: Writing – review & editing. ST: Writing – review & editing. CC: Writing – review & editing. DA: Writing – review & editing.
